# 3,3′-[1,2-Phenyl­enebis(methyl­ene)]bis­(1-ethyl­benzimidazolium) dibromide

**DOI:** 10.1107/S1600536812002802

**Published:** 2012-02-04

**Authors:** Rosenani A. Haque, Muhammad Adnan Iqbal, Srinivasa Budagumpi, Madhukar Hemamalini, Hoong-Kun Fun

**Affiliations:** a School of Chemical Sciences, Universiti Sains Malaysia, 11800 USM, Penang, Malaysia; b X-ray Crystallography Unit, School of Physics, Universiti Sains Malaysia, 11800 USM, Penang, Malaysia

## Abstract

In the title mol­ecular salt, C_26_H_28_N_4_^2+^·2Br^−^, the central benzene ring makes dihedral angles of 76.75 (11) and 82.40 (10)° with the pendant benzimidazole rings. The corresponding angle between the benzimidazole rings is 57.03 (9)°. In the crystal, the cations and anions are linked *via* C—H⋯Br hydrogen bonds, forming sheets lying parallel to the *bc* plane. The crystal structure also features weak C—H⋯π inter­actions.

## Related literature

For background to the biological activities of benzimidazole compounds, see: Mohan *et al.* (2011 ▶).
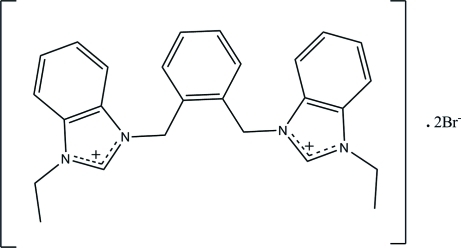


## Experimental

### 

#### Crystal data

C_26_H_28_N_4_^2+^·2Br^−^
*M* *_r_* = 556.34
Monoclinic, 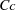

*a* = 9.7093 (7) Å
*b* = 35.796 (3) Å
*c* = 8.0340 (6) Å
β = 118.230 (1)°
*V* = 2460.1 (3) Å^3^
*Z* = 4
Mo *K*α radiation
μ = 3.32 mm^−1^
*T* = 296 K
0.45 × 0.32 × 0.23 mm

#### Data collection

Bruker APEXII DUO CCD diffractometer
Absorption correction: multi-scan (*SADABS*; Bruker, 2009 ▶) *T*_min_ = 0.318, *T*_max_ = 0.516
16606 measured reflections
8158 independent reflections
6244 reflections with *I* > 2σ(*I*)
*R*_int_ = 0.023

#### Refinement

*R*[*F* ^2^ > 2σ(*F* ^2^)] = 0.033
*wR*(*F* ^2^) = 0.080
*S* = 1.02
8158 reflections
291 parameters
2 restraints
H-atom parameters constrained
Δρ_max_ = 0.71 e Å^−3^
Δρ_min_ = −0.28 e Å^−3^
Absolute structure: Flack (1983 ▶), 3727 Friedel pairs
Flack parameter: 0.009 (6)

### 

Data collection: *APEX2* (Bruker, 2009 ▶); cell refinement: *SAINT* (Bruker, 2009 ▶); data reduction: *SAINT*; program(s) used to solve structure: *SHELXTL* (Sheldrick, 2008 ▶); program(s) used to refine structure: *SHELXTL*; molecular graphics: *SHELXTL*; software used to prepare material for publication: *SHELXTL* and *PLATON* (Spek, 2009 ▶).

## Supplementary Material

Crystal structure: contains datablock(s) global, I. DOI: 10.1107/S1600536812002802/hb6611sup1.cif

Structure factors: contains datablock(s) I. DOI: 10.1107/S1600536812002802/hb6611Isup2.hkl

Supplementary material file. DOI: 10.1107/S1600536812002802/hb6611Isup3.cml

Additional supplementary materials:  crystallographic information; 3D view; checkCIF report

## Figures and Tables

**Table 1:** Hydrogen-bond geometry (Å, °) *Cg*4 is the centroid of the C11–C16 ring.

*D*—H⋯*A*	*D*—H	H⋯*A*	*D*⋯*A*	*D*—H⋯*A*
C9—H9*A*⋯Br2	0.93	2.76	3.610 (2)	152
C10—H10*A*⋯Br1^i^	0.97	2.92	3.884 (2)	171
C10—H10*B*⋯Br2	0.97	2.91	3.820 (2)	156
C15—H15*A*⋯Br2^ii^	0.93	2.80	3.700 (3)	163
C17—H17*B*⋯Br2	0.97	2.78	3.696 (3)	158
C24—H24*A*⋯Br1	0.93	2.73	3.579 (2)	152
C5—H5*A*⋯*Cg*4^iii^	0.93	2.92	3.630 (3)	135

Symmetry codes: (i) 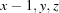; (ii) 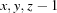; (iii) 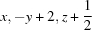.

**Fig. 1.**
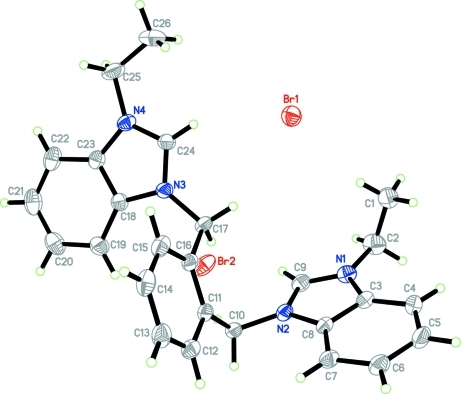
The asymmetric unit of the title compound, showing 30% probability displacement ellipsoids.

**Fig. 2.**
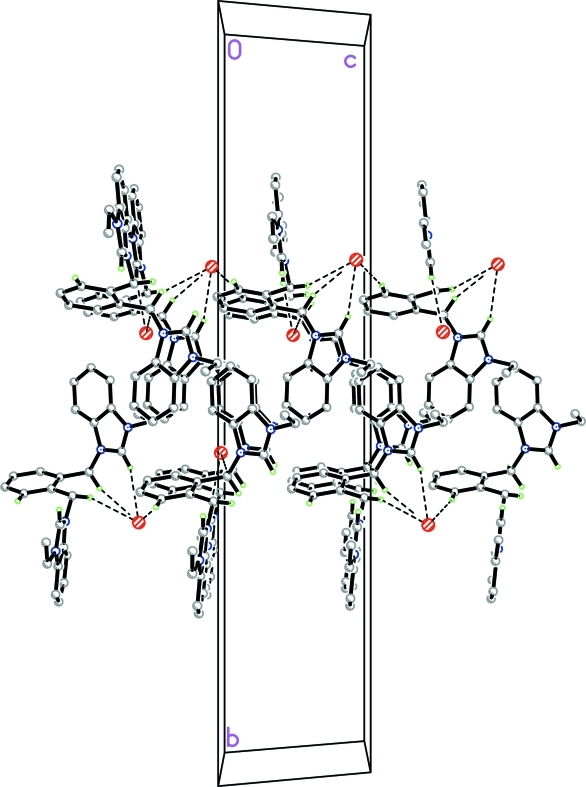
The crystal packing of the title compound.

## supplementary crystallographic information

### Comment

Many benzimidazole containing compounds are biologically active (Mohan *et al.*, 2011). As part of our studies in this area, we now describe the title compound, (I).

The central benzene ring (C11–C16) makes dihedral angles of 76.75 (11) and 82.40 (10)° with the adjacent benzimidazole rings (N1,N2/C3–C9) and (N3,N4/C18–C24). The dihedral angle between the benzimidazole rings (N1,N2/C3–C9) and (N1,N2/C3–C9) is 57.03 (9)°. The benzimidazole (N1,N2/C3–C9) and (N3,N4/C18–C24) rings are approximately planar [maximum deviations of 0.020 (3) Å for atom C3 and 0.014 (2) Å for atom N3, respectively].

In the crystal (Fig. 2), the cations and anions are linked *via* C—H···Br (Table 1) hydrogen bonds, forming two-dimensional networks lying parallel to the the *bc*-plane. The crystal structure also features weak C—H···π interactions involving the centroid of the phenyl (C11–C16) ring.

### Experimental

A mixture of benzimidazole (2.36 g, 20 mmol) and finely ground potassium hydroxide (2.36 g, 30 mmol) in 30 ml of DMSO was stirred at room temperature (27–28 °C) for 30 min. 1-Bromoethane (1.50 ml, 20 mmol) was added drop wise in this consistently stirring mixture and further stirred for 2 h at same temperature, poured into water (300 ml) and was extracted by chloroform (5 × 20 ml). The extract was dried by magnesium sulphate and evaporated under reduced pressure to get *N*-ethylbenzimidazole as a thick yellowish fluid (2.52 g, 86.30%). Furthermore, a mixture of 1 (1.46 g, 10 mmol) and 1,2-bis(bromomethyl)benzene (1.32 g, 5 mmol) in dioxane (30 ml) was refluxed at 110 °C for 18 h. Desired compound (2.2Br) appeared as beige–colored precipitates in dark brown solution. The mixture was filtered and precipitates were washed by fresh dioxane (3 × 5 ml), dried at room temperature for 24 h, and soft lumps so obtained were ground to fine powder (2.40 g, 86.33%). Saturated solution of 2.2Br in methanol (0.5 ml) was exposed to diethyl ether vapours (vapour diffusion) at room temperature overnight to get colourless blocks of (I).

### Refinement

All hydrogen atoms were positioned geometrically [C–H = 0.93–0.97 Å] and were refined using a riding model, with *U*_iso_(H) = 1.2 or 1.5 *U*_eq_(C). A rotating group model was applied to the methyl groups. 3727 Friedel pairs were used to determine the absolute structure. One outliner, (4 0 -4), was omitted in the final refinement.

### Crystal data

**Table:**

C_26_H_28_N_4_^2+^·2Br^−^	*F*(000) = 1128
*M**_r_* = 556.34	*D*_x_ = 1.502 Mg m^−^^3^
Monoclinic, *C**c*	Mo *K*α radiation, λ = 0.71073 Å
Hall symbol: C -2yc	Cell parameters from 5440 reflections
*a* = 9.7093 (7) Å	θ = 2.9–27.3°
*b* = 35.796 (3) Å	µ = 3.32 mm^−^^1^
*c* = 8.0340 (6) Å	*T* = 296 K
β = 118.230 (1)°	Block, colourless
*V* = 2460.1 (3) Å^3^	0.45 × 0.32 × 0.23 mm
*Z* = 4	

### Data collection

**Table:**

Bruker APEXII DUO CCD diffractometer	8158 independent reflections
Radiation source: fine-focus sealed tube	6244 reflections with *I* > 2σ(*I*)
Graphite monochromator	*R*_int_ = 0.023
φ and ω scans	θ_max_ = 32.5°, θ_min_ = 2.5°
Absorption correction: multi-scan (*SADABS*; Bruker, 2009)	*h* = −14→14
*T*_min_ = 0.318, *T*_max_ = 0.516	*k* = −54→42
16606 measured reflections	*l* = −12→12

### Refinement

**Table:**

Refinement on *F*^2^	Secondary atom site location: difference Fourier map
Least-squares matrix: full	Hydrogen site location: inferred from neighbouring sites
*R*[*F*^2^ > 2σ(*F*^2^)] = 0.033	H-atom parameters constrained
*wR*(*F*^2^) = 0.080	*w* = 1/[σ^2^(*F*_o_^2^) + (0.*P*)^2^ + 0.1437*P*] where *P* = (*F*_o_^2^ + 2*F*_c_^2^)/3
*S* = 1.02	(Δ/σ)_max_ = 0.003
8158 reflections	Δρ_max_ = 0.71 e Å^−^^3^
291 parameters	Δρ_min_ = −0.28 e Å^−^^3^
2 restraints	Absolute structure: Flack (1983), 3727 Friedel pairs
Primary atom site location: structure-invariant direct methods	Flack parameter: 0.009 (6)

### Special details

**Table:**

Geometry. All s.u.'s (except the s.u. in the dihedral angle between two l.s. planes) are estimated using the full covariance matrix. The cell s.u.'s are taken into account individually in the estimation of s.u.'s in distances, angles and torsion angles; correlations between s.u.'s in cell parameters are only used when they are defined by crystal symmetry. An approximate (isotropic) treatment of cell s.u.'s is used for estimating s.u.'s involving l.s. planes.
Refinement. Refinement of F^2^ against ALL reflections. The weighted R-factor wR and goodness of fit S are based on F^2^, conventional R-factors R are based on F, with F set to zero for negative F^2^. The threshold expression of F^2^ > 2σ(F^2^) is used only for calculating R-factors(gt) etc. and is not relevant to the choice of reflections for refinement. R-factors based on F^2^ are statistically about twice as large as those based on F, and R- factors based on ALL data will be even larger.

### Fractional atomic coordinates and isotropic or equivalent isotropic displacement parameters (Å^2^)

**Table:**

	*x*	*y*	*z*	*U*_iso_*/*U*_eq_	
Br1	0.68944 (3)	0.918762 (6)	0.48259 (3)	0.05182 (7)	
Br2	0.38351 (4)	0.822199 (8)	0.90664 (4)	0.06457 (9)	
N1	0.4060 (2)	0.95348 (6)	0.8359 (3)	0.0441 (4)	
N2	0.2014 (2)	0.92236 (5)	0.6352 (3)	0.0378 (4)	
N3	0.4142 (2)	0.82699 (5)	0.4234 (3)	0.0386 (4)	
N4	0.5754 (2)	0.79004 (5)	0.3842 (3)	0.0416 (4)	
C1	0.6769 (3)	0.97225 (8)	0.9292 (4)	0.0569 (7)	
H1A	0.7799	0.9729	1.0354	0.085*	
H1B	0.6509	0.9964	0.8706	0.085*	
H1C	0.6739	0.9541	0.8395	0.085*	
C2	0.5611 (3)	0.96170 (10)	0.9949 (4)	0.0615 (7)	
H2A	0.5990	0.9399	1.0759	0.074*	
H2B	0.5519	0.9820	1.0690	0.074*	
C3	0.3020 (3)	0.97982 (6)	0.7130 (3)	0.0405 (4)	
C4	0.3092 (3)	1.01828 (7)	0.7066 (4)	0.0503 (6)	
H4A	0.3959	1.0316	0.7930	0.060*	
C5	0.1818 (4)	1.03575 (7)	0.5662 (5)	0.0578 (7)	
H5A	0.1826	1.0616	0.5568	0.069*	
C6	0.0502 (3)	1.01593 (7)	0.4361 (4)	0.0542 (6)	
H6A	−0.0333	1.0290	0.3424	0.065*	
C7	0.0416 (3)	0.97753 (7)	0.4438 (4)	0.0453 (5)	
H7A	−0.0461	0.9643	0.3592	0.054*	
C8	0.1711 (2)	0.95996 (6)	0.5847 (3)	0.0372 (4)	
C9	0.3416 (3)	0.92020 (6)	0.7867 (3)	0.0433 (5)	
H9A	0.3878	0.8981	0.8494	0.052*	
C10	0.0966 (2)	0.89051 (6)	0.5423 (3)	0.0388 (4)	
H10A	−0.0035	0.8952	0.5378	0.047*	
H10B	0.1408	0.8682	0.6174	0.047*	
C11	0.0711 (3)	0.88369 (5)	0.3442 (3)	0.0372 (4)	
C12	−0.0774 (3)	0.89068 (6)	0.1938 (4)	0.0485 (5)	
H12A	−0.1560	0.8994	0.2187	0.058*	
C13	−0.1095 (3)	0.88490 (7)	0.0100 (4)	0.0537 (6)	
H13A	−0.2082	0.8903	−0.0886	0.064*	
C14	0.0046 (4)	0.87111 (7)	−0.0274 (4)	0.0537 (6)	
H14A	−0.0173	0.8670	−0.1518	0.064*	
C15	0.1523 (3)	0.86321 (6)	0.1182 (4)	0.0478 (5)	
H15A	0.2289	0.8538	0.0912	0.057*	
C16	0.1865 (3)	0.86927 (5)	0.3048 (3)	0.0368 (4)	
C17	0.3497 (3)	0.86073 (6)	0.4620 (4)	0.0411 (5)	
H17A	0.4179	0.8817	0.4767	0.049*	
H17B	0.3461	0.8576	0.5798	0.049*	
C18	0.3557 (3)	0.79088 (6)	0.4066 (3)	0.0406 (5)	
C19	0.2223 (3)	0.77759 (8)	0.4100 (4)	0.0525 (6)	
H19A	0.1533	0.7933	0.4260	0.063*	
C20	0.1996 (4)	0.73945 (9)	0.3877 (5)	0.0635 (8)	
H20A	0.1112	0.7292	0.3865	0.076*	
C21	0.3028 (4)	0.71617 (7)	0.3674 (5)	0.0665 (8)	
H21A	0.2825	0.6906	0.3547	0.080*	
C22	0.4359 (4)	0.72922 (7)	0.3652 (4)	0.0581 (7)	
H22A	0.5060	0.7133	0.3523	0.070*	
C23	0.4582 (3)	0.76776 (6)	0.3837 (3)	0.0404 (4)	
C24	0.5441 (3)	0.82501 (6)	0.4069 (3)	0.0406 (5)	
H24A	0.6047	0.8455	0.4108	0.049*	
C25	0.7016 (3)	0.77625 (9)	0.3487 (5)	0.0621 (7)	
H25A	0.6559	0.7646	0.2251	0.074*	
H25B	0.7602	0.7573	0.4418	0.074*	
C26	0.8105 (5)	0.80600 (13)	0.3563 (8)	0.0893 (12)	
H26A	0.8782	0.7964	0.3099	0.134*	
H26B	0.8720	0.8142	0.4845	0.134*	
H26C	0.7518	0.8267	0.2795	0.134*	

### Atomic displacement parameters (Å^2^)

**Table:**

	*U*^11^	*U*^22^	*U*^33^	*U*^12^	*U*^13^	*U*^23^
Br1	0.05117 (12)	0.04447 (12)	0.05944 (14)	0.00068 (11)	0.02585 (11)	0.00191 (11)
Br2	0.0914 (2)	0.05391 (15)	0.06103 (16)	0.03112 (14)	0.04636 (15)	0.01796 (12)
N1	0.0401 (9)	0.0531 (11)	0.0364 (9)	0.0054 (8)	0.0158 (8)	0.0004 (8)
N2	0.0395 (9)	0.0323 (8)	0.0399 (9)	0.0074 (7)	0.0174 (8)	0.0014 (7)
N3	0.0384 (9)	0.0314 (8)	0.0517 (10)	0.0024 (7)	0.0261 (9)	0.0025 (7)
N4	0.0413 (10)	0.0384 (9)	0.0485 (10)	0.0098 (7)	0.0240 (8)	0.0037 (8)
C1	0.0458 (13)	0.0531 (14)	0.0583 (16)	0.0057 (11)	0.0136 (13)	0.0064 (11)
C2	0.0454 (13)	0.085 (2)	0.0391 (13)	0.0005 (14)	0.0076 (11)	−0.0018 (12)
C3	0.0437 (11)	0.0399 (10)	0.0417 (11)	0.0040 (9)	0.0234 (9)	−0.0029 (8)
C4	0.0533 (13)	0.0392 (11)	0.0636 (15)	−0.0033 (10)	0.0319 (12)	−0.0091 (10)
C5	0.0717 (17)	0.0332 (11)	0.0821 (19)	0.0063 (11)	0.0475 (16)	0.0009 (11)
C6	0.0530 (13)	0.0404 (12)	0.0704 (17)	0.0135 (11)	0.0301 (13)	0.0089 (11)
C7	0.0398 (11)	0.0415 (11)	0.0506 (13)	0.0100 (9)	0.0182 (10)	0.0021 (9)
C8	0.0398 (10)	0.0326 (9)	0.0416 (11)	0.0065 (8)	0.0213 (9)	0.0005 (8)
C9	0.0447 (11)	0.0423 (11)	0.0413 (11)	0.0091 (9)	0.0192 (10)	0.0043 (8)
C10	0.0378 (10)	0.0335 (10)	0.0506 (12)	0.0034 (8)	0.0253 (10)	0.0000 (8)
C11	0.0411 (10)	0.0255 (8)	0.0473 (11)	0.0003 (7)	0.0229 (9)	−0.0004 (7)
C12	0.0438 (12)	0.0378 (11)	0.0579 (14)	0.0073 (9)	0.0191 (11)	−0.0024 (10)
C13	0.0534 (14)	0.0394 (11)	0.0517 (14)	0.0041 (11)	0.0112 (12)	−0.0014 (10)
C14	0.0760 (18)	0.0378 (11)	0.0438 (13)	−0.0009 (12)	0.0253 (13)	−0.0006 (9)
C15	0.0660 (15)	0.0352 (10)	0.0530 (13)	0.0019 (10)	0.0370 (12)	−0.0005 (9)
C16	0.0444 (11)	0.0245 (8)	0.0466 (11)	0.0015 (8)	0.0257 (9)	0.0028 (7)
C17	0.0425 (11)	0.0352 (10)	0.0523 (13)	0.0060 (9)	0.0279 (10)	−0.0028 (9)
C18	0.0440 (11)	0.0339 (10)	0.0459 (11)	−0.0011 (8)	0.0229 (10)	0.0035 (8)
C19	0.0524 (14)	0.0476 (13)	0.0659 (16)	−0.0074 (11)	0.0348 (13)	0.0019 (11)
C20	0.0695 (18)	0.0507 (15)	0.0742 (18)	−0.0191 (14)	0.0372 (15)	0.0068 (13)
C21	0.088 (2)	0.0359 (12)	0.0665 (17)	−0.0097 (14)	0.0293 (17)	0.0035 (12)
C22	0.0764 (19)	0.0329 (11)	0.0570 (15)	0.0096 (12)	0.0249 (14)	0.0031 (10)
C23	0.0451 (11)	0.0337 (9)	0.0393 (11)	0.0056 (9)	0.0174 (9)	0.0034 (8)
C24	0.0373 (10)	0.0367 (10)	0.0523 (13)	0.0014 (8)	0.0249 (10)	0.0022 (9)
C25	0.0527 (14)	0.0677 (17)	0.0768 (19)	0.0179 (13)	0.0396 (14)	−0.0038 (14)
C26	0.065 (2)	0.100 (3)	0.133 (3)	−0.0016 (19)	0.071 (2)	−0.011 (3)

### Geometric parameters (Å, º)

**Table:**

N1—C9	1.316 (3)	C10—H10B	0.9700
N1—C3	1.393 (3)	C11—C12	1.397 (3)
N1—C2	1.472 (3)	C11—C16	1.399 (3)
N2—C9	1.332 (3)	C12—C13	1.373 (4)
N2—C8	1.396 (3)	C12—H12A	0.9300
N2—C10	1.474 (3)	C13—C14	1.369 (5)
N3—C24	1.331 (3)	C13—H13A	0.9300
N3—C18	1.393 (3)	C14—C15	1.385 (4)
N3—C17	1.460 (3)	C14—H14A	0.9300
N4—C24	1.321 (3)	C15—C16	1.391 (3)
N4—C23	1.388 (3)	C15—H15A	0.9300
N4—C25	1.469 (3)	C16—C17	1.519 (3)
C1—C2	1.499 (5)	C17—H17A	0.9700
C1—H1A	0.9600	C17—H17B	0.9700
C1—H1B	0.9600	C18—C23	1.372 (3)
C1—H1C	0.9600	C18—C19	1.392 (4)
C2—H2A	0.9700	C19—C20	1.381 (4)
C2—H2B	0.9700	C19—H19A	0.9300
C3—C4	1.381 (3)	C20—C21	1.371 (5)
C3—C8	1.394 (3)	C20—H20A	0.9300
C4—C5	1.368 (4)	C21—C22	1.382 (5)
C4—H4A	0.9300	C21—H21A	0.9300
C5—C6	1.402 (4)	C22—C23	1.393 (3)
C5—H5A	0.9300	C22—H22A	0.9300
C6—C7	1.380 (4)	C24—H24A	0.9300
C6—H6A	0.9300	C25—C26	1.481 (5)
C7—C8	1.383 (3)	C25—H25A	0.9700
C7—H7A	0.9300	C25—H25B	0.9700
C9—H9A	0.9300	C26—H26A	0.9600
C10—C11	1.510 (3)	C26—H26B	0.9600
C10—H10A	0.9700	C26—H26C	0.9600
			
C9—N1—C3	108.49 (19)	C13—C12—C11	121.3 (3)
C9—N1—C2	126.0 (2)	C13—C12—H12A	119.3
C3—N1—C2	125.5 (2)	C11—C12—H12A	119.3
C9—N2—C8	107.76 (18)	C14—C13—C12	119.6 (2)
C9—N2—C10	125.46 (17)	C14—C13—H13A	120.2
C8—N2—C10	126.77 (18)	C12—C13—H13A	120.2
C24—N3—C18	107.65 (19)	C13—C14—C15	120.7 (3)
C24—N3—C17	125.96 (19)	C13—C14—H14A	119.6
C18—N3—C17	126.3 (2)	C15—C14—H14A	119.6
C24—N4—C23	107.7 (2)	C14—C15—C16	120.1 (2)
C24—N4—C25	127.8 (2)	C14—C15—H15A	119.9
C23—N4—C25	124.4 (2)	C16—C15—H15A	119.9
C2—C1—H1A	109.5	C15—C16—C11	119.6 (2)
C2—C1—H1B	109.5	C15—C16—C17	119.2 (2)
H1A—C1—H1B	109.5	C11—C16—C17	121.2 (2)
C2—C1—H1C	109.5	N3—C17—C16	111.95 (19)
H1A—C1—H1C	109.5	N3—C17—H17A	109.2
H1B—C1—H1C	109.5	C16—C17—H17A	109.2
N1—C2—C1	112.0 (2)	N3—C17—H17B	109.2
N1—C2—H2A	109.2	C16—C17—H17B	109.2
C1—C2—H2A	109.2	H17A—C17—H17B	107.9
N1—C2—H2B	109.2	C23—C18—C19	122.5 (2)
C1—C2—H2B	109.2	C23—C18—N3	106.5 (2)
H2A—C2—H2B	107.9	C19—C18—N3	131.0 (2)
C4—C3—N1	132.0 (2)	C20—C19—C18	115.4 (3)
C4—C3—C8	121.7 (2)	C20—C19—H19A	122.3
N1—C3—C8	106.29 (19)	C18—C19—H19A	122.3
C5—C4—C3	116.4 (2)	C21—C20—C19	122.4 (3)
C5—C4—H4A	121.8	C21—C20—H20A	118.8
C3—C4—H4A	121.8	C19—C20—H20A	118.8
C4—C5—C6	122.2 (2)	C20—C21—C22	122.4 (2)
C4—C5—H5A	118.9	C20—C21—H21A	118.8
C6—C5—H5A	118.9	C22—C21—H21A	118.8
C7—C6—C5	121.6 (2)	C21—C22—C23	115.6 (3)
C7—C6—H6A	119.2	C21—C22—H22A	122.2
C5—C6—H6A	119.2	C23—C22—H22A	122.2
C6—C7—C8	116.0 (2)	C18—C23—N4	107.35 (18)
C6—C7—H7A	122.0	C18—C23—C22	121.7 (3)
C8—C7—H7A	122.0	N4—C23—C22	130.9 (2)
C7—C8—C3	122.1 (2)	N4—C24—N3	110.8 (2)
C7—C8—N2	131.4 (2)	N4—C24—H24A	124.6
C3—C8—N2	106.51 (18)	N3—C24—H24A	124.6
N1—C9—N2	110.93 (19)	N4—C25—C26	113.3 (3)
N1—C9—H9A	124.5	N4—C25—H25A	108.9
N2—C9—H9A	124.5	C26—C25—H25A	108.9
N2—C10—C11	112.86 (18)	N4—C25—H25B	108.9
N2—C10—H10A	109.0	C26—C25—H25B	108.9
C11—C10—H10A	109.0	H25A—C25—H25B	107.7
N2—C10—H10B	109.0	C25—C26—H26A	109.5
C11—C10—H10B	109.0	C25—C26—H26B	109.5
H10A—C10—H10B	107.8	H26A—C26—H26B	109.5
C12—C11—C16	118.6 (2)	C25—C26—H26C	109.5
C12—C11—C10	118.1 (2)	H26A—C26—H26C	109.5
C16—C11—C10	123.2 (2)	H26B—C26—H26C	109.5
			
C9—N1—C2—C1	108.0 (3)	C14—C15—C16—C17	−179.4 (2)
C3—N1—C2—C1	−72.7 (3)	C12—C11—C16—C15	1.7 (3)
C9—N1—C3—C4	177.2 (3)	C10—C11—C16—C15	178.9 (2)
C2—N1—C3—C4	−2.3 (4)	C12—C11—C16—C17	−179.5 (2)
C9—N1—C3—C8	−0.8 (3)	C10—C11—C16—C17	−2.3 (3)
C2—N1—C3—C8	179.8 (2)	C24—N3—C17—C16	119.9 (3)
N1—C3—C4—C5	−178.5 (3)	C18—N3—C17—C16	−63.6 (3)
C8—C3—C4—C5	−0.8 (4)	C15—C16—C17—N3	−38.6 (3)
C3—C4—C5—C6	0.4 (4)	C11—C16—C17—N3	142.5 (2)
C4—C5—C6—C7	0.6 (5)	C24—N3—C18—C23	1.2 (3)
C5—C6—C7—C8	−1.1 (4)	C17—N3—C18—C23	−175.9 (2)
C6—C7—C8—C3	0.8 (4)	C24—N3—C18—C19	−178.7 (3)
C6—C7—C8—N2	178.7 (3)	C17—N3—C18—C19	4.2 (4)
C4—C3—C8—C7	0.2 (4)	C23—C18—C19—C20	−0.3 (4)
N1—C3—C8—C7	178.4 (2)	N3—C18—C19—C20	179.6 (3)
C4—C3—C8—N2	−178.2 (2)	C18—C19—C20—C21	1.2 (5)
N1—C3—C8—N2	0.0 (3)	C19—C20—C21—C22	−0.9 (5)
C9—N2—C8—C7	−177.4 (3)	C20—C21—C22—C23	−0.4 (5)
C10—N2—C8—C7	1.1 (4)	C19—C18—C23—N4	179.1 (2)
C9—N2—C8—C3	0.8 (3)	N3—C18—C23—N4	−0.8 (3)
C10—N2—C8—C3	179.3 (2)	C19—C18—C23—C22	−1.0 (4)
C3—N1—C9—N2	1.4 (3)	N3—C18—C23—C22	179.0 (2)
C2—N1—C9—N2	−179.2 (2)	C24—N4—C23—C18	0.1 (3)
C8—N2—C9—N1	−1.4 (3)	C25—N4—C23—C18	−175.1 (2)
C10—N2—C9—N1	−179.9 (2)	C24—N4—C23—C22	−179.7 (3)
C9—N2—C10—C11	−114.9 (2)	C25—N4—C23—C22	5.0 (4)
C8—N2—C10—C11	66.8 (3)	C21—C22—C23—C18	1.4 (4)
N2—C10—C11—C12	−111.6 (2)	C21—C22—C23—N4	−178.8 (3)
N2—C10—C11—C16	71.3 (2)	C23—N4—C24—N3	0.7 (3)
C16—C11—C12—C13	−2.3 (3)	C25—N4—C24—N3	175.7 (3)
C10—C11—C12—C13	−179.6 (2)	C18—N3—C24—N4	−1.2 (3)
C11—C12—C13—C14	1.7 (4)	C17—N3—C24—N4	175.9 (2)
C12—C13—C14—C15	−0.5 (4)	C24—N4—C25—C26	5.3 (5)
C13—C14—C15—C16	0.0 (4)	C23—N4—C25—C26	179.6 (3)
C14—C15—C16—C11	−0.6 (3)		

### Hydrogen-bond geometry (Å, º)

Cg4 is the centroid of the C11–C16 ring.

**Table:**

*D*—H···*A*	*D*—H	H···*A*	*D*···*A*	*D*—H···*A*
C9—H9*A*···Br2	0.93	2.76	3.610 (2)	152
C10—H10*A*···Br1^i^	0.97	2.92	3.884 (2)	171
C10—H10*B*···Br2	0.97	2.91	3.820 (2)	156
C15—H15*A*···Br2^ii^	0.93	2.80	3.700 (3)	163
C17—H17*B*···Br2	0.97	2.78	3.696 (3)	158
C24—H24*A*···Br1	0.93	2.73	3.579 (2)	152
C5—H5*A*···*Cg*4^iii^	0.93	2.92	3.630 (3)	135

Symmetry codes: (i) *x*−1, *y*, *z*; (ii) *x*, *y*, *z*−1; (iii) *x*, −*y*+2, *z*+1/2.

## References

[bb1] Bruker (2009). *APEX2*, *SAINT* and *SADABS* Bruker AXS Inc., Madison, Wisconsin, USA.

[bb2] Flack, H. D. (1983). *Acta Cryst.* A**39**, 876–881.

[bb3] Mohan, V. G., Sreenivasulu, N., Rao, A. S. & Chigiri, S. (2011). *Der Pharma Chem.* **3**, 446–452.

[bb4] Sheldrick, G. M. (2008). *Acta Cryst.* A**64**, 112–122. 10.1107/S0108767307043930 18156677

[bb5] Spek, A. L. (2009). *Acta Cryst.* D**65**, 148–155. 10.1107/S090744490804362X PMC2631630 19171970

